# Sex-disaggregated age-standardized crude suicide rates in Sierra Leone, 2000–2019

**DOI:** 10.1093/inthealth/ihaf137

**Published:** 2025-11-25

**Authors:** Umaru Sesay, Augustus Osborne

**Affiliations:** Workforce Unit, African Field Epidemiology Network, P.O. Box 12874, Freetown, Sierra Leone; Division of NCD, Injuries, and Mental Health, Africa Centres for Disease Control and Prevention, Addis Ababa, P.O. Box 3243, Ethiopia; Research Unit, Institute for Development, P.O. Box 1313, Freetown, Sierra Leone

**Keywords:** inequality, mental health, public health, sex disparities, Sierra Leone, suicide

## Abstract

**Background:**

This study aims to determine the prevalence and sex-based disparities in age-standardized crude suicide rates in Sierra Leone from 2000 to 2019.

**Methods:**

This study employed a time-trend design utilizing nationally representative data on age-standardized crude suicide rates (per 100 000 population) from the WHO Health Equity Assessment Toolkit. The primary outcome measure was the age-standardized crude suicide rate, disaggregated by sex. Inequality measures, including difference, ratio, population attributable fraction and population attributable risk, were calculated to assess disparities between males and females.

**Results:**

From 2000 to 2019, the age-standardized crude suicide rate in Sierra Leone increased from 5.7 to 6.7 per 100 000 population, with notable fluctuations observed. Significant sex-based disparities were evident throughout the study period, with males consistently exhibiting higher suicide rates than females. The absolute difference in suicide rates between males and females narrowed slightly, from −4.0 per 100 000 in 2000 to −3.3 per 100 000 in 2019. However, the ratio of male-to-female suicide rates increased from 0.5 in 2000 to 0.6 in 2019.

**Conclusions:**

This study highlights persistent sex-based disparities in age-standardized crude suicide rates in Sierra Leone, disproportionately affecting males. This study recommends strengthening mental health services and integrating them into primary healthcare systems.

## Introduction

Suicide, the intentional act of ending one’s own life, represents a significant public health challenge that affects individuals across all age groups.^1^ Certain populations are at heightened risk, including those with substance use disorders,^2^ those with mental disorders,^3^ the socially deprived and poor,^4^ as well as victims of intimate partner violence, interpersonal violence, sexual violence and child trauma.^5^

Globally, approximately 800 000 individuals die by suicide each year,^6^ with a significant proportion of these fatalities occurring among adolescents aged 15–19 y.^7^ From 2000 to 2022, suicide rates increased by 26%.^1^ In 2022 alone, an estimated 12.8 million people thought about suicide, 3.7 million planned it and 1.5 million attempted it.^1^ By 2023, suicide was identified as the second leading cause of death among individuals aged 10–34 y and the eighth leading cause among those aged 10–64 y.^1^ Furthermore, the financial burden of suicide was estimated at US$490 billion in 2019, accounting for medical expenses, lost productivity and diminished quality of life.^6^

In response to the rising suicide crisis, global leaders have endorsed international treaties, including the inclusion of suicide prevention in the United Nations Sustainable Development Goal 3.4.2.^8^ The WHO has also promoted initiatives such as the Thirteenth General Programme of Work^9^ and the Comprehensive Mental Health Action Plan.^10^ Despite these international efforts, suicide remains a pressing public health issue, disproportionately impacting low- and middle-income countries, which account for 77% of global suicides.^11^ In Africa, while the age-standardized crude suicide rate appears relatively low,^12^ it is likely that many cases are under-reported due to weak health systems, particularly in countries such as Sierra Leone.

Sierra Leone, classified as a low-income country and ranked 184 out of 193 on the Human Development Index in 2022, has experienced numerous traumatic health crises, including a civil war, an Ebola outbreak, social and political unrest and economic instability. These factors have significantly contributed to rising suicide rates in the country. In 2020, Sierra Leone was ranked 56th out of 184 countries in terms of suicide mortality, with a rate of 11.25 per 100 000 population.^13^ The following year, the suicide crude rate was reported at 5.09 per 100 000 population, with males at 6.91 per 100 000 and females at 3.29 per 100 000.^14^

To combat this growing public health issue, the government of Sierra Leone, through the Ministry of Health, established the Directorate of Mental Health and Non-Communicable Diseases in 2017,^15^ which includes suicide prevention. Despite these initiatives, suicide continues to affect thousands of individuals within the population. A study on the epidemiology of suicidal behaviors among school-going adolescents in post-conflict Sierra Leone found that one in six children reported suicidal ideation, and two in 10 adolescents had attempted suicide in 2017.^16^ Another study conducted in the same year reported a prevalence of suicidal ideation at 14.6%, with 14.2% having made a plan and 19% having attempted suicide.^16^ While these investigations have yielded valuable insights to inform evidence-based interventions aimed at reducing the suicide burden, there remains a lack of comprehensive studies examining sex-disaggregated age-standardized crude suicide rates in Sierra Leone. This study seeks to address this critical knowledge gap by generating evidence-based findings to inform public health strategies aimed at reducing the burden of suicide and addressing inequalities in suicide rates among males and females. The objective of this research is to analyze the disparities in age-standardized crude suicide rates by sex in Sierra Leone from 2000 to 2019.

## Materials and methods

### Study design and period

This study employed a time-trend design to analyze the age-standardized crude suicide rates in Sierra Leone, from 2000 to 2019.

### Data source

In this study, we extracted all available data points from 2000 to 2019 on the crude rates of suicide (per 100 000 population) from the WHO’s Health Equity Assessment Toolkit (HEAT).^17^ The WHO HEAT platform is a publicly accessible data repository, incorporating various data sources, including the Institute of Health Metrics and Evaluation Global Burden of Disease dataset, which was utilized in our analysis. This platform (WHO HEAT) ensures that the data are preprocessed and nationally representative, adhering to rigorous data processing and quality assurance protocols that promote consistency and comparability across different data points. A comprehensive assessment was performed to address missing data, employing imputation methods or exclusion strategies based on the nature and extent of the missing information. Our quality assurance measures included validation processes that assessed both the completeness and accuracy of the data. This involved cross-referencing the extracted data with national health statistics and demographic surveys. Additionally, the WHO employed statistical techniques to identify and rectify anomalies or inconsistencies within the dataset. Prior to analysis, the data underwent age standardization to account for variations in age distribution across populations. It is important to note that no additional weighting was applied beyond the standardization process performed by the WHO HEAT.

### Outcome measure and dimension of inequality

The primary outcome measure employed in this study was the age-standardized crude suicide rates, reported per 100 000 population. This metric was chosen for its capacity to adjust for variations in population age structures, thereby enabling meaningful comparisons across different time periods and among subgroups. The dimension of inequality examined in this research was sex, with the population divided into two distinct categories: male and female. Although the HEAT platform offers the capability to analyze various dimensions of inequality, including socioeconomic status, geographic region and urban-rural residence, this study specifically focused on sex-based disparities. This decision was guided by the limitations inherent in the available data and the central aim of the investigation. The analysis aimed to clarify the differences in age-standardized crude suicide rates between sexes, which represent a critical public health concern in Sierra Leone, particularly given the disproportionate impact of such incidents on males. By collectively examining these populations, this study seeks to highlight the interconnectedness of health issues related to suicide, ultimately contributing to the formulation of more effective public health strategies and interventions tailored to address these disparities.

### Data analysis

R software version 4.1.0 (R development Core Team, Vienna, Australia) was used for analysis.^18^ To streamline the data cleaning and analysis process, custom scripts were developed in R, specifically designed to automate several critical tasks:

#### Data importation

Scripts were created to efficiently import data from the WHO HEAT database, ensuring the capture of all relevant variables.

#### Data cleaning

Custom functions were implemented to check for missing values, identify outliers and standardize variable formats. This automation significantly improved the efficiency and reproducibility of the data cleaning process.

#### Age standardization

Dedicated scripts were employed to perform age standardization of crude rates, facilitating accurate comparisons across various age groups. Age-standardization techniques were employed to adjust for alterations in population structure and growth. This enabled us to compare crude rates across different years while controlling for variations in age distribution.

The decision to use R and develop custom scripts was motivated by the need for flexibility and precision in our analysis.

Data analysis utilized the WHO HEAT software, which facilitates the computation of estimates, CIs and summary measures of inequality. The HEAT platform offers tools for generating both absolute and relative measures of inequality, enabling a comprehensive exploration of disparities. Four key metrics were employed to assess sex-based disparities in age-standardized crude suicide rates: difference (D), ratio (R), population attributable fraction (PAF) and population attributable risk (PAR).

#### Difference

This metric quantifies the absolute disparity in crude rates between females and males by directly comparing their values, providing a clear indication of the magnitude of inequality in absolute terms.

#### Ratio

This metric calculates the proportional difference in crude rates by dividing the crude rate among females by that of males, offering a relative measure of the disparity.

#### Population attributable fraction

This metric represents the overall crude attributable to sex-based inequality, reflecting the potential reduction in crude if the disparity were eliminated.

#### Population attributable risk

This metric indicates the excess age-standardized crude suicide rates attributable to sex-based inequality in the population, providing an absolute measure of the burden.

Collectively, these metrics offered a nuanced understanding of the magnitude and context of sex-based disparities in crude suicide rates. It is crucial to highlight that negative values for PAF and PAR indicate that addressing health disparities could result in a decrease in crude suicide rates. Specifically, these negative values imply that certain population groups experience a lower crude suicide rate than would be anticipated if disparities were eliminated. In the realm of public health interventions, such negative PAF and PAR values signify a substantial opportunity for health improvements by prioritizing the reduction of disparities, underscoring the necessity for equitable health policies that focus on vulnerable populations. This insight also aids policymakers in effective resource allocation, ensuring that interventions are directed toward populations that would derive the greatest benefit from diminished disparities.^19–21^ Estimates and 95% CIs were computed for both male and female subgroups across each year of analysis. The results are presented in both tabular and graphical formats to enhance interpretation and to emphasize trends over time. For additional information on the inequality measures employed, readers are directed to the relevant literature.^19–21^

## Results

### Age-standardized crude suicide rates (per 100 000 population) in Sierra Leone

**Figure 1.**
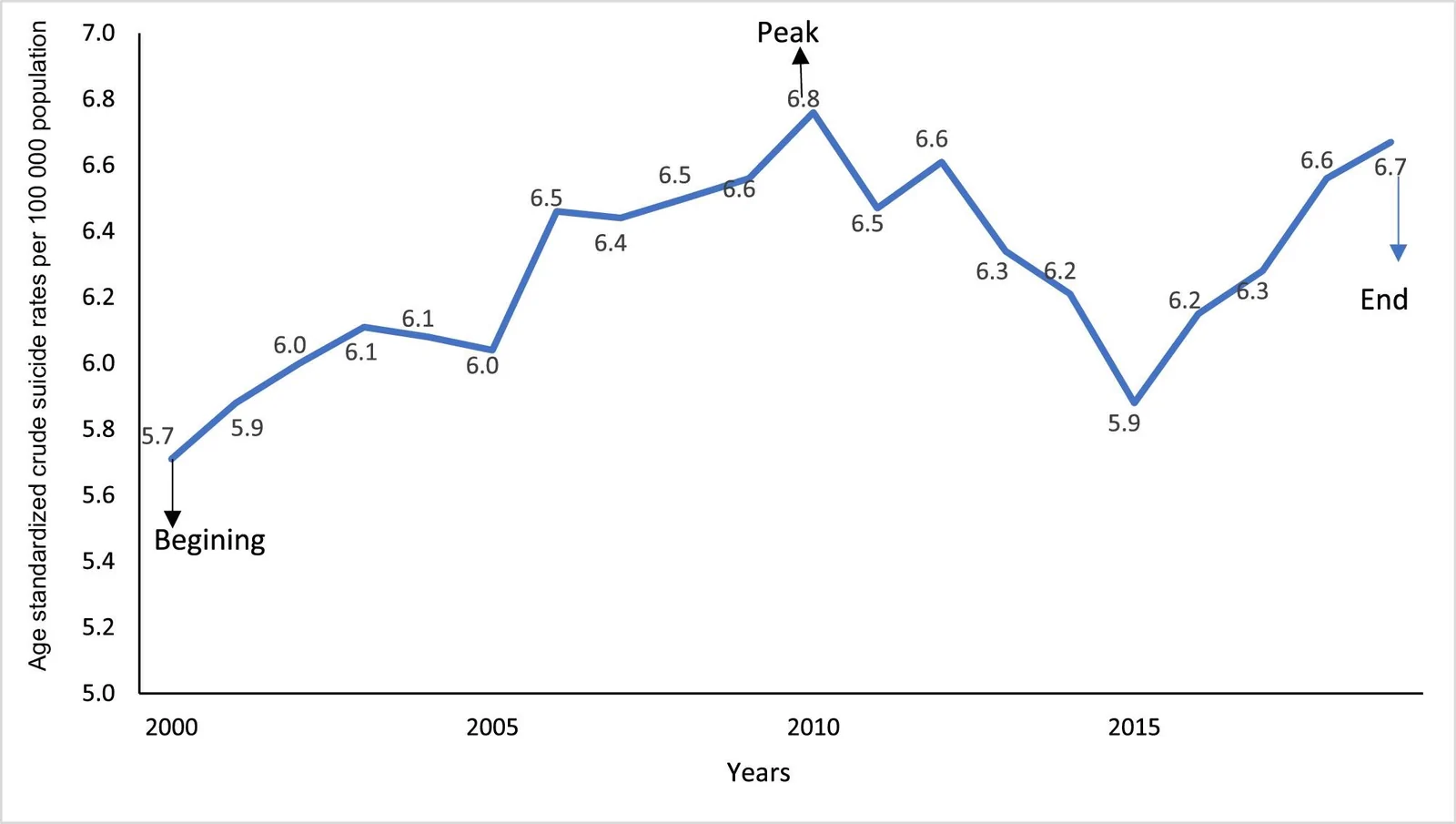
Trends of age standardized crude suicide rates in Sierra Leone, 2000–2019.

Figure 1 shows a notable increase in age-standardized crude suicide rates in Sierra Leone from 5.7 per 100 000 population in 2000 to 6.7 per 100 000 population in 2019. Age-standardized crude suicide rates fluctuated over time. From 2000 to 2005, a gradual increase in age-standardized crude suicide rates was observed, rising by 5.26%, and from 2005 to 2010 there was a further increase by 13.3%. However, from 2010 to 2015, the age-standardized crude suicide rates decreased by 13.24%. From 2015 to 2019, the age-standardized crude suicide rates increased again by 13.56%, almost at the same rate as that observed from 2005 to 2010. The lowest age-standardized crude suicide rate was 5.7 per 100 000 population in 2000, and then there was a continuous upward trend, with a peak in 2010 at an age-standardized crude suicide rate of 6.8 per 100 000 population.

Table 1 presents significant sex-based disparities in age-standardized crude suicide rates throughout the study period, with males consistently exhibiting higher crude rates than females. Overall, there was a gradual increase in age-standardized crude suicide rates, with the lowest rate reported in 2000 and the highest in 2019. For instance, in 2000, the age-standardized crude suicide rate was 3.7 per 100 000 population for females and 7.7 per 100 000 population for males, which increased steadily until 2010, with females recording 4.8 per 100 000 population and males 8.7 per 100 000 population. After that, there was a gradual decrease in age-standardized crude suicide rates for females until 2018, reaching a rate of 4.9 per 100 000 population, and subsequently increasing to 5.1 per 100 000 population in 2019. Similar patterns were displayed for males, decreasing gradually until 2015 and attaining a rate of 7.6 per 100 000 population, then starting to increase slowly, attaining a rate of 8.3 per 100 000 population in 2019.

**Table 1.** Trends of sex-disaggregated suicide rates in Sierra Leone, 2000–2019

Year	Subgroup	Estimate	LB	UB
2000	Female	3.7	2.2	6.0
2000	Male	7.7	4.0	13.1
2001	Female	3.9	2.3	6.3
2001	Male	7.9	4.1	13.3
2002	Female	4.1	2.3	6.5
2002	Male	8.0	4.1	13.5
2003	Female	4.2	2.4	6.7
2003	Male	8.1	4.1	13.6
2004	Female	4.1	2.3	6.6
2004	Male	8.1	4.1	13.7
2005	Female	4.1	2.3	6.7
2005	Male	8.0	4.1	13.5
2006	Female	4.3	2.5	7.1
2006	Male	8.6	4.4	14.5
2007	Female	4.4	2.5	7.2
2007	Male	8.5	4.4	14.3
2008	Female	4.5	2.5	7.3
2008	Male	8.6	4.4	14.3
2009	Female	4.6	2.6	7.5
2009	Male	8.6	4.5	14.3
2010	Female	4.8	2.7	7.9
2010	Male	8.7	4.6	14.5
2011	Female	4.7	2.7	7.7
2011	Male	8.3	4.4	13.7
2012	Female	4.8	2.7	7.8
2012	Male	8.5	4.5	13.9
2013	Female	4.6	2.6	7.5
2013	Male	8.1	4.4	13.5
2014	Female	4.4	2.5	7.2
2014	Male	8.0	4.3	13.5
2015	Female	4.2	2.4	7.0
2015	Male	7.6	4.1	12.7
2016	Female	4.5	2.5	7.6
2016	Male	7.8	4.2	13.1
2017	Female	4.7	2.6	7.8
2017	Male	7.9	4.2	13.2
2018	Female	4.9	2.7	8.2
2018	Male	8.2	4.4	13.8
2019	Female	5.1	2.8	8.5
2019	Male	8.3	4.5	13.9

LB: lower bound; UB: upper bound.

Note: blue represents male and white represents female.

### Sex inequality of age-standardized crude suicide rates in Sierra Leone, 2000–2019

Table 2 presents inequality measures related to age-standardized crude suicide rates in Sierra Leone, disaggregated by sex (male vs female) from 2000 to 2019. The difference (D) in age-standardized crude suicide rates between males and females exhibited a slight increase, rising from -4 per 100 000 population in 2000 to -3 per 100 000 population in 2019, indicating a narrowing gap in age-standardized crude suicide rates between the sexes. Despite this narrowing difference, both the PAF and PAR remained constant at 0% throughout the study period. This constancy suggests that no suicides in the population could be attributed to specific risk factors under consideration, indicating that these risk factors did not significantly influence the crude rates of suicide during this time frame. Additionally, the ratio (R) of crude rates between males and females increased slightly, from 0.5 in 2000 to 0.6 in 2019.

**Table 2.** Inequality of sex-disaggregated suicide rates in Sierra Leone, 2000–2019

Year	Dimension	Measure	Estimate
2000	Sex	D	−4.0
2000	Sex	PAF	0.0
2000	Sex	PAR	0.0
2000	Sex	R	0.5
2001	Sex	D	−4.0
2001	Sex	PAF	0.0
2001	Sex	PAR	0.0
2001	Sex	R	0.5
2002	Sex	D	−3.9
2002	Sex	PAF	0.0
2002	Sex	PAR	0.0
2002	Sex	R	0.5
2003	Sex	D	−3.9
2003	Sex	PAF	0.0
2003	Sex	PAR	0.0
2003	Sex	R	0.5
2004	Sex	D	−4.1
2004	Sex	PAF	0.0
2004	Sex	PAR	0.0
2004	Sex	R	0.5
2005	Sex	D	−3.9
2005	Sex	PAF	0.0
2005	Sex	PAR	0.0
2005	Sex	R	0.5
2006	Sex	D	−4.2
2006	Sex	PAF	0.0
2006	Sex	PAR	0.0
2006	Sex	R	0.5
2007	Sex	D	−4.1
2007	Sex	PAF	0.0
2007	Sex	PAR	0.0
2007	Sex	R	0.5
2008	Sex	D	−4.1
2008	Sex	PAF	0.0
2008	Sex	PAR	0.0
2008	Sex	R	0.5
2009	Sex	D	−4.0
2009	Sex	PAF	0.0
2009	Sex	PAR	0.0
2009	Sex	R	0.5
2010	Sex	D	−3.9
2010	Sex	PAF	0.0
2010	Sex	PAR	0.0
2010	Sex	R	0.5
2011	Sex	D	−3.6
2011	Sex	PAF	0.0
2011	Sex	PAR	0.0
2011	Sex	R	0.6
2012	Sex	D	−3.7
2012	Sex	PAF	0.0
2012	Sex	PAR	0.0
2012	Sex	R	0.6
2013	Sex	D	−3.6
2013	Sex	PAF	0.0
2013	Sex	PAR	0.0
2013	Sex	R	0.6
2014	Sex	D	−3.6
2014	Sex	PAF	0.0
2014	Sex	PAR	0.0
2014	Sex	R	0.5
2015	Sex	D	−3.3
2015	Sex	PAF	0.0
2015	Sex	PAR	0.0
2015	Sex	R	0.6
2016	Sex	D	−3.2
2016	Sex	PAF	0.0
2016	Sex	PAR	0.0
2016	Sex	R	0.6
2017	Sex	D	−3.2
2017	Sex	PAF	0.0
2017	Sex	PAR	0.0
2017	Sex	R	0.6
2018	Sex	D	−3.3
2018	Sex	PAF	0.0
2018	Sex	PAR	0.0
2018	Sex	R	0.6
2019	Sex	D	−3.3
2019	Sex	PAF	0.0
2019	Sex	PAR	0.0
2019	Sex	R	0.6

D: difference; PAF: population attributable fraction; PAR: population attributable risk; R: ratio.

## Discussion

This study examined trends in age-standardized crude suicide rates, disaggregated by sex, in Sierra Leone, from 2000 to 2019. The findings revealed a dynamic pattern of age-standardized crude suicide rates over the two decades, with an overall increase from 5.7 per 100 000 population in 2000 to 6.7 per 100 000 population in 2019. The results also highlighted significant sex-based disparities, with males consistently exhibiting higher suicide rates compared with females throughout the study period. The difference in age-standardized crude suicide rates between males and females narrowed slightly over time, from -4.0 per 100 000 population in 2000 to -3.3 per 100 000 population in 2019. Despite this narrowing gap, the PAF and PAR remained constant at 0%, suggesting that the underlying risk factors considered in this study did not significantly influence the observed trends in age-standardized crude suicide rates. These results underscore the importance of ongoing monitoring and targeted public health interventions to address the complex factors contributing to suicide in Sierra Leone.

The trends in age-standardized crude suicide rates in Sierra Leone reveal important insights into the public health burden of suicide in the country. From 2000 to 2005, there was a gradual increase in the age-standardized crude suicide rate of 5.26%. This rise can primarily be attributed to the consequent aftermath of the prolonged civil war that occurred from 1991 to 2002.^22^ During this tumultuous period, a substantial portion of the population was subjected to extreme poverty, developed mental health disorders, lacked access to quality healthcare services and faced numerous human rights violations.^22^ The interplay of these factors likely contributed to the observed increase in age-standardized crude suicide rates during this time frame. Furthermore, the increase noted from 2005 to 2010 can be linked to several compounding factors. The lingering effects of the civil war were exacerbated by political instability, particularly the tensions surrounding the nationwide presidential and parliamentary elections in 2007.^22^ Additionally, heightened inflation of essential commodities and the absence of dedicated suicide-prevention legislation may have further influenced this upward trend.^23^ Contrarily, the decrease in age-standardized crude suicide rates from 2010 to 2015 was unexpected, especially considering that this period encompassed significant public health crises, including the Ebola outbreak from 2014 to 2016 and a cholera outbreak in 2012. Potential explanations for this decline may include the under-reporting of suicide incidents and the prioritization of governmental efforts to manage and control these outbreaks, which may have inadvertently compromised the collection of suicide data. Also, the launch of mental health policy in 2012 could have also garnered momentum on suicide-prevention interventions, thereby resulting in the observed decrease.^24^

Despite the observed decrease from 2010 to 2015, alarmingly, the country experienced a sudden increase in age-standardized crude suicide rates from 2015 to 2019. This resurgence underscores the need for continued attention to mental health issues and the implementation of effective suicide-prevention strategies in Sierra Leone. These findings align with global trends, where suicide rates often fluctuate due to various factors, including economic instability, social upheaval and access to mental health services.^25^ For example, countries in sub-Saharan Africa have reported similar patterns of fluctuating suicide rates, with peaks often coinciding with periods of political instability or economic crises.^26^

A key finding of this study is the persistent disparity in age-standardized crude suicide rates between males and females, with males consistently exhibiting higher age-standardized crude suicide rates across all years of the study period. Although the absolute difference between male and female suicide rates narrowed slightly over time, the ratio of male-to-female suicide rates remained relatively stable, disproportionately affecting males. Several factors contribute to the higher age-standardized crude suicide rates observed among males compared with females in Sierra Leone. One significant reason is the pressure on men to serve as the sole providers for their families, particularly in rural areas where economic opportunities are limited. This pressure can lead to feelings of inadequacy and depression, which often increase their risk of suicidal behavior. Additionally, societal norms that dictate male dominance and discourage vulnerability further exacerbate mental health issues among men. The expectation that males should not express weakness or openly discuss their mental health struggles prevents them from seeking help and support. This cultural stigma surrounding male emotional expression contributes to the deterioration of mental health and consequently results in incidence of suicide. The interplay of these factors elucidates the elevated age-standardized crude suicide rates among males in Sierra Leone, highlighting the urgent need for targeted interventions that address the unique challenges faced by men in this context. This finding is consistent with global patterns, where males are typically at greater risk of suicide compared with females.^27^ This disparity has been attributed to several factors, including differences in the methods used for suicide, with males more likely to use lethal means, as well as gender differences in help-seeking behavior and mental health stigma.^28^ The limited availability of mental health services in Sierra Leone may disproportionately affect men, as they are less likely to seek help for mental health issues compared with women.^29^

The findings of this study are particularly relevant in the context of sub-Saharan Africa, where suicide remains a significant public health challenge. The age-standardized crude suicide rates observed in Sierra Leone are comparable to those reported in other countries in the region, such as Uganda and Kenya, which have reported rates ranging from 5 to 10 per 100 000 population in recent years.^30,31^ However, the trends in Sierra Leone also reflect unique socio-political and economic factors, including the legacy of the civil war, ongoing poverty and limited access to healthcare services.

Mental health services in sub-Saharan Africa are generally under-resourced, with many countries allocating <1% of their health budgets to mental health.^32^ In Sierra Leone, the situation is further compounded by a lack of trained mental health professionals and inadequate infrastructure for mental healthcare delivery. These challenges underscore the need for targeted interventions to address the underlying determinants of suicide, including poverty, unemployment and social isolation, as well as efforts to improve access to mental health services.

## Strength and limitations

The strengths of the current study include its use of WHO HEAT data, which provide credibility and ensure comparability with international studies. Also, the study's findings are framed around policy and health system implications, which makes it valuable for policymakers. Although this study provides valuable insights into trends and disparities in suicide rates in Sierra Leone, several limitations should be acknowledged. First, the study relied on age-standardized crude suicide rates, which may not fully capture the complexity of suicide trends in the population. Age-standardized rates could provide a more accurate comparison across different demographic groups. Second, the study did not examine the underlying causes or risk factors contributing to suicide, such as mental health disorders, substance use or socioeconomic factors. Future research should explore these factors to better understand the drivers of suicide in Sierra Leone. Third, the data used in this study may be subject to under-reporting and misclassification, which are common challenges in suicide research, particularly in low-resource settings. Cultural stigma and inadequate death registration systems may result in an underestimation of suicide rates, particularly among women and marginalized populations. Efforts to improve the accuracy and completeness of suicide data in Sierra Leone are essential for informing effective prevention strategies. Fourth, although the study utilized a two-decade dataset, the most recent data available are from 2019. This limitation may hinder our ability to provide comprehensive evidence regarding the current prevalence of suicide in Sierra Leone. The absence of data beyond 2019 restricts our analysis to the years prior, thereby potentially overlooking more recent trends. Fifth, the authors were unable to verify the observed findings with local data due to data limitations. Finally, the study did not account for potential regional variations in suicide rates within Sierra Leone. Given the country’s diverse socioeconomic and cultural landscape, regional differences in suicide rates and risk factors may exist. Future studies should investigate these variations to identify high-risk areas and inform targeted interventions.

### Future directions

Building on the findings of this study, future research should focus on identifying the socioeconomic, cultural and psychological factors contributing to suicide in Sierra Leone. Longitudinal studies and qualitative research could provide a deeper understanding of the lived experiences of individuals at risk of suicide and the barriers they face in accessing mental healthcare. Additionally, efforts to improve mental health literacy and reduce stigma around suicide are critical for promoting help-seeking behavior and preventing suicide in the population. Policy-oriented research is also needed to evaluate the impact of existing mental health policies and programs in Sierra Leone and to identify opportunities for improvement. For example, integrating mental health services into primary healthcare settings could help address the treatment gap and ensure that individuals at risk of suicide receive timely and appropriate care. Finally, regional and international collaborations could facilitate the sharing of best practices and resources for suicide prevention in sub-Saharan Africa.

### Conclusion

This study highlights the dynamic nature of suicide trends in Sierra Leone over two decades, with an overall increase in age-standardized crude suicide rates and persistent sex-based disparities. The findings underscore the urgent need for targeted public health interventions such as gender-sensitive mental health education, community-based support networks and culturally appropriate outreach strategies for men to address the underlying determinants of suicide and improve access to mental health services. In the context of sub-Saharan Africa, where mental health resources are limited, these results provide valuable insights for policymakers and healthcare providers seeking to reduce the burden of suicide in the region. By prioritizing mental health on the national agenda and investing in evidence-based prevention strategies, Sierra Leone can take meaningful steps toward reducing suicide rates and improving the well-being of its population.

## Data Availability

The dataset used can be accessed at https://www.who.int/data/inequality-monitor/assessment_toolkit.

## References

[1] CDC . Facts about suicide [Internet]. Suicide Prevention. 2025. Available from: https://www.cdc.gov/suicide/facts/index.html [accessed 10 June 2025].

[2] Padmanathan P, Hall K, Moran P, et al. Prevention of suicide and reduction of self-harm among people with substance use disorder: A systematic review and meta-analysis of randomised controlled trials. Compr Psychiatry. 2020;96:152135. 31810026 10.1016/j.comppsych.2019.152135

[3] Melo APS, Dippenaar IN, Johnson SC, et al. All-cause and cause-specific mortality among people with severe mental illness in Brazil’s public health system, 2000-15: a retrospective study. Lancet Psychiatry. 2022;9(10):771–81. 35964638 10.1016/S2215-0366(22)00237-1 PMC9477749

[4] Lee H, Park CHK, Rhee SJ, et al. The influence of poverty attribution on attitudes toward suicide and suicidal thought: a cross-national comparison between South Korean, Japanese, and American populations. Compr Psychiatry. 2021;109:152259. 34273607 10.1016/j.comppsych.2021.152259

[5] Ásgeirsdóttir HG, Valdimarsdóttir UA, Þorsteinsdóttir ÞK, et al. The association between different traumatic life events and suicidality. Eur J Psychotraumatol. 2018;9(1):1510279. 30220981 10.1080/20008198.2018.1510279 PMC6136384

[6] An S, Lim S, Kim HW, et al. Global prevalence of suicide by latitude: A systematic review and meta-analysis. Asian J Psychiatr. 2023;81:103454. 36634498 10.1016/j.ajp.2023.103454 PMC9822839

[7] WHO . Suicide key facts [Internet]. 2025. Available from: https://www.who.int/news-room/fact-sheets/detail/suicide [accessed 10 June 2025].

[8] United Nations . The 17 Sustainable Development Goals [Internet]. Available from: https://sdgs.un.org/goals [accessed 10 June 2025].

[9] WHO . Thirteenth General Programme of Work 2019–2023: promote health, keep the world safe, serve the vulnerable [Internet]. 2019. Available from: https://www.who.int/publications/i/item/WHO-PRP-18.1 [accessed 10 June 2025].

[10] WHO . Comprehensive Mental Health Action Plan 2013-2030 [Internet]. 2021. Available from: https://www.who.int/publications/i/item/9789240031029 [accessed 10 June 2025].

[11] Renaud J, MacNeil SL, Vijayakumar L, et al. Suicidal ideation and behavior in youth in low- and middle-income countries: A brief review of risk factors and implications for prevention. Front Psychiatry. 2022;13:1044354. 36561636 10.3389/fpsyt.2022.1044354 PMC9763724

[12] Mars B, Burrows S, Hjelmeland H, et al. Suicidal behaviour across the African continent: a review of the literature. BMC Public Health. 2014;14(1):606. 24927746 10.1186/1471-2458-14-606 PMC4067111

[13] World Life Expectancy . Suicide in Sierra Leone [Internet]. World Life Expectancy. 2020. Available from: https://www.worldlifeexpectancy.com/fr/sierra-leone-suicide [accessed 10 June 2025].

[14] World Population Review . Suicide rate by country [Internet]. worldpopulationreview.com. 2025. Available from: https://worldpopulationreview.com/country-rankings/suicide-rate-by-country#countries-with-the-highest-suicide-rates-per-100k [accessed 10 June 2025].

[15] Ministry of Health, Government of Sierra Leone . Non-communicable Disease Policy. Sierra Leone: Government of Sierra Leone. 2020.

[16] Oppong Asante K, Quarshie EN, Onyeaka HK. Epidemiology of suicidal behaviours amongst school-going adolescents in post-conflict Sierra Leone. J Affect Disord. 2021;295:1. 34706473 10.1016/j.jad.2021.08.147

[17] WHO . Health Equity Assessment Toolkit [Internet]. 2025. Available from: https://www.who.int/data/inequality-monitor/assessment_toolkit [accessed 12 June 2025].

[18] Kalibera T, Meyer S, Hornik K. Changes in R 4.0–4.1. The R Journal. 2021;13(1):638–40.

[19] Hesseinpoor AR, Nambiar D, Schlotheuber A, et al. Health Equity Assessment Toolkit (HEAT): software for exploring and comparing health inequalities in countries | BMC Medical Research Methodology | Full Text [Internet]. 2016. Available from: https://bmcmedresmethodol.biomedcentral.com/articles/10.1186/s12874-016-0229-9 [accessed 12 June 2025]. 10.1186/s12874-016-0229-9 PMC5069829 27760520

[20] Schlotheuber A, Hosseinpoor AR. Summary Measures of health inequality: a review of existing measures and their application. Int J Environ Res Public Health. 2022;19(6):3697. 35329383 10.3390/ijerph19063697 PMC8992138

[21] WHO . Handbook on health inequality monitoring with a special focus on low- and middle-income countries. 2013. Available from: https://www.who.int/publications/i/item/9789241548632 [accessed 12 June 2025].

[22] Betancourt TS, Keegan K, Farrar J, et al. The intergenerational impact of war on mental health and psychosocial wellbeing: lessons from the longitudinal study of war-affected youth in Sierra Leone. Confl Health. 2020;14(1):62. 32884581 10.1186/s13031-020-00308-7 PMC7461150

[23] Harris D, Endale T, Lind UH, et al. Mental health in Sierra Leone. B J Psych Int. 2020;17(1):14–6. 10.1192/bji.2019.17 PMC8277533 34287429

[24] Sierra Leone Mental Health Policy Launched by First Lady [Internet]. 2012. Available from: https://mentalhealthcoalitionsl.com/2012/10/15/mh-policy-launc/ [accessed 15 June 2025].

[25] Lund C, Brooke-Sumner C, Baingana F, et al. Social determinants of mental disorders and the Sustainable Development Goals: a systematic review of reviews. Lancet Psychiatry. 2018;5(4):357–69. 29580610 10.1016/S2215-0366(18)30060-9

[26] Claveria O . Global economic uncertainty and suicide: worldwide evidence. Soc Sci Med. 2022;305:115041. 35598442 10.1016/j.socscimed.2022.115041

[27] Naghavi M , Global Burden of Disease self-Harm Collaborators. Global, regional, and national burden of suicide mortality 1990 to 2016: systematic analysis for the Global Burden of Disease Study 2016. BMJ. 2019;364:l94. 31339847 10.1136/bmj.l94 PMC6598639

[28] Chang Q, Yip PSF, Chen YY. Gender inequality and suicide gender ratios in the world. J Affect Disord. 2019;243:297–304. 30261445 10.1016/j.jad.2018.09.032

[29] Harris D, Endale T, Lind UH, et al. Mental health in Sierra Leone. B J Psych Int. 2020;17(1):14–6. 10.1192/bji.2019.17 PMC8277533 34287429

[30] Ongeri L, Larsen DA, Jenkins R, et al. Community suicide rates and related factors within a surveillance platform in Western Kenya. BMC Psychiatry. 2022;22(1):7. 34983463 10.1186/s12888-021-03649-6 PMC8729019

[31] Ongeri L, Nyawira M, Kariuki SM, et al. Perspectives on reasons for suicidal behaviour and recommendations for suicide prevention in Kenya: qualitative study. B J Psych Open. 2023;9(2):e38. 10.1192/bjo.2023.7 PMC9970164 36797822

[32] Freeman M . Investing for population mental health in low and middle income countries-where and why? Int J Ment Health Syst. 2022;16(1):38. 35953845 10.1186/s13033-022-00547-6 PMC9366832

